# The New Nitinol Conformable Self-Expandable Metal Stents for Malignant Colonic Obstruction: A Pilot Experience as Bridge to Surgery Treatment

**DOI:** 10.1155/2014/651765

**Published:** 2014-01-02

**Authors:** Roberto Di Mitri, Filippo Mocciaro

**Affiliations:** Gastroenterology and Endoscopy Unit, A.R.N.A.S. Civico-Di Cristina-Benfratelli Hospital, Piazza Leotta No. 4, 90100 Palermo, Italy

## Abstract

*Introduction*. Self-expandable metal stents (SEMS) are a nonsurgical option for treatment of malignant colorectal obstruction also as a bridge to surgery approach. The new nitinol conformable stent has improved clinical outcomes in these kinds of patients. We report a pilot experience with nitinol conformable SEMS placement as bridge to surgery treatment in patients with colorectal obstruction. *Materials and Methods*. Between April and August 2012, we collected data on colonic nitinol conformable SEMS placement in a cohort of consecutive symptomatic patients, with malignant colorectal obstruction, who were treated as a bridge to surgery. Technical success, clinical success, and adverse events were recorded. *Results.* Ten patients (7 male (70%)), with a mean age of 69.2 ± 10.1, were evaluated. The mean length of the stenosis was 3.6 ± 0.6 cm. Five patients (50%) were treated on an emergency basis. The median time from stent placement to surgery was 16 days (interquartile range 7–21). Technical and clinical success was achieved in all patients with a significant early improvement of symptoms. No adverse events due to the SEMS placement were observed. *Conclusion*. This pilot study confirmed the important role of nitinol conformable SEMS as a bridge to surgery option in the treatment of symptomatic malignant colorectal obstruction.

## 1. Introduction

Gastrointestinal self-expandable metal stents (SEMS) placement is an affective endoscopic technique as a nonsurgical approach in the treatment of malignant colonic obstruction [1]. Up to 20% of patients with colorectal cancer (CRC) experience an acute symptomatic obstruction [2, 3]. In addition, considering the high incidence of CRC, SEMS placement has to be considered a valid endoscopic treatment not only in patients with incurable malignant obstruction but also as a bridge to surgery [2]. Moreover, SEMS placement can be a rescue therapy in those with acute severe colonic obstruction with significant improvement of overall quality of life (QoL) and QoL related to gastrointestinal symptoms [4]. Nevertheless, 20–30% of patients experience early or late SEMS-related complications, such as migration, obstruction, or perforation [5–7]. The advent of new nickel-titanium (nitinol) conformable stents with a shape memory alloy has improved the treatment of malignant biliary and gastrointestinal strictures [8]. The aim of this study is to report a pilot experience on the use of nitinol SEMS as bridge to surgery treatment in patients with CRC.

## 2. Patient Characteristics, Study Design, and Stenting Technique

**Figure 1:**
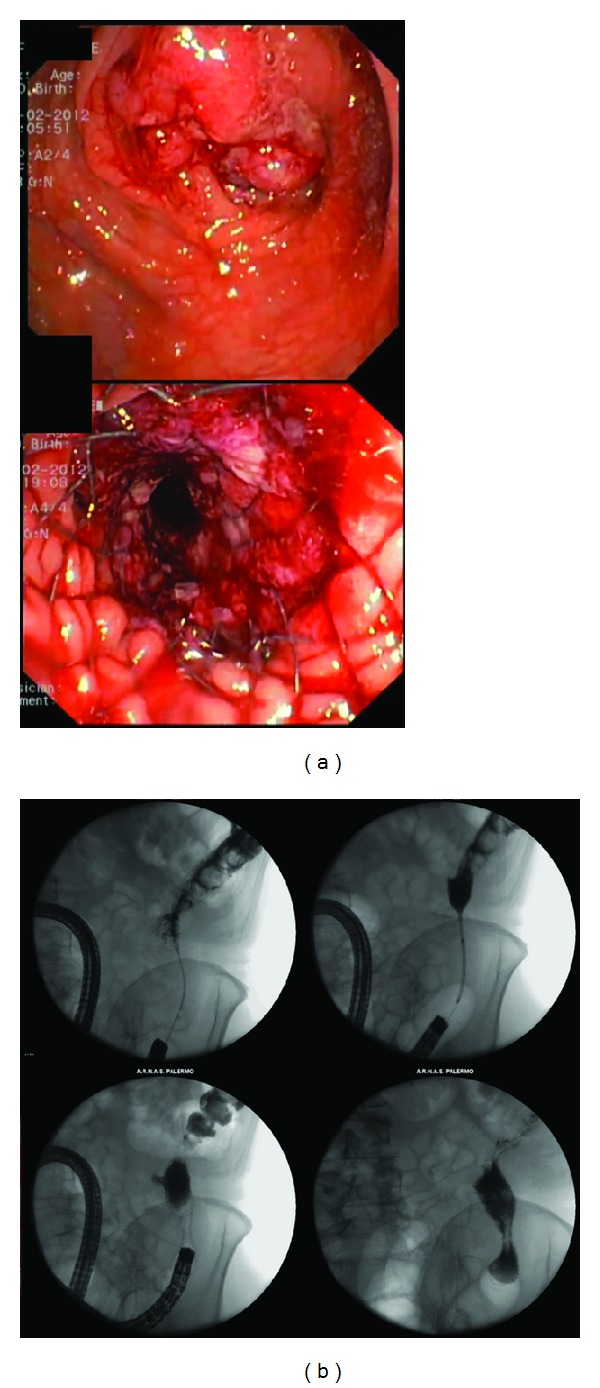
Procedure for a nitinol SEMS placement in a 78-year-old man admitted because of intestinal occlusion due to colorectal cancer of the descending colon (a). The patient's symptoms improved early after stent placement (b).

Between April and August 2012, we prospectively collected data on patients treated with placement of Niti-S enteral uncovered stent (D-Type) (TaeWoong, Seoul, Korea) due to obstructive CRC (Figure 1(a)). All patients were diagnosed as having CRC obstruction by computed tomography (CT) scan and/or colonoscopy with biopsy. Indication for SEMS placement was a symptomatic obstruction: patients who are unable to pass stool and gas, are vomiting, and are having abdominal pain and paradoxical diarrhea.

All patients were hospitalized and underwent a complete blood count and chemistry and coagulation parameters. The bridge to surgery approach was discussed with the surgeon in all treated patients taking into account both the cancer characteristics/location and the overall clinical condition. Emergency nitinol SEMS placement was also performed with the aid of an anesthesiologist. An informed consent was always obtained before the procedure informing hospital IRB. When possible, the patients were prepared with cleansing enemas to facilitate stricture visualization and further stent insertion.

The nitinol conformable SEMS were placed under endoscopic guidance according to manufacturers' instruction by an experienced endoscopist (RDM, over 140 SEMS placed) with the aid of fluoroscopy (Figure 1(b)). All placed SEMS were uncovered and all procedures took on average 30 minutes.

Data on demographics, technical success, clinical efficacy, and complications were collected in an electronic database and subsequently exported to the statistical software for the final analysis.

## 3. Definitions

Technical success was defined as the successful placement of the nitinol SEMS through the stenosis and confirmed by radiological markers. After 24 and 48 hours all treated patients underwent abdominal X-ray in order to exclude early major complications and to evaluate the complete opening of the SEMS.

Clinical success was defined as significant colonic decompression within 72 hours after SEMS placement and associated with improvement of QoL (resolution of symptoms and oral intake of food).

Emergency SEMS placement was performed as rescue therapy in patients admitted to the emergency room with acute severe symptomatic malignant colonic obstruction detected by CT scan.

Perforation, early stent obstruction, or migration was considered a major complication. Each death was investigated as to whether it was related to the SEMS placement.

## 4. Statistical Analyses

Data were analyzed using the SPSS 15 (SPSS Inc., Chicago, IL, USA) software package. Continuous variables were summarized as mean (±standard deviation (SD)) or median (range) according to their distribution. Categorical variables were summarized as frequency and percentage. We considered the following variables: demographics, site and extension of neoplasia, indication (elective or emergency procedure), technical success, clinical success with QoL improvement, and adverse events.

## 5. Results

We collected data on 10 patients (7 male (70%)), with a mean age of 69.2 ± 10.1. All patient characteristics are summarized in Table 1. Site of colorectal obstruction was rectosigmoid in 5 patients, descending colon in 2, splenic flexure in 1, transverse colon in 1, and ascending colon in 1. The mean length of the stenosis was 3.6 ± 0.6 cm. Five patients (50%) were treated on an emergency basis, while the remaining underwent elective treatment. All patients underwent SEMS placement as a bridge to surgery. The median time from stent placement to surgery was 16 days (interquartile range, 7–21). After SEMS placement, technical success was achieved in all patients with a significant improvement of symptoms and QoL within 24 to 48 hours. Decompression of the colon was confirmed by X-ray of the abdomen.

During surgery, the stent was observed to be in situ in all patients. The tumor could be resected in all cases and stents were removed en bloc with the tumor.

## 6. Safety

None procedural or postprocedural complications were recorded as well as postsurgical complications. No early stents migration was recorded. No colonic perforation was noted at surgical inspection.

## 7. Discussion

Our pilot study shows that endoscopic nitinol conformable SEMS placement is a safe and effective bridge treatment in patients with symptomatic CRC who are waiting for surgery. Previous studies have reported the effectiveness of SEMS as a nonsurgical approach for the relief of obstructive symptoms in patients with CRC (89–96% clinical success) [9–12].

In operable patients, SEMS placement can be an effective therapeutic treatment before resective surgery. In a recent study on the role of SEMS as a bridge to surgery, a procedural success rate of 98% (177/181) was reported, with 94% clinical success before elective surgery [13], and these data where in accordance to previous data were the bridge to surgery approach ranged between 83% and 100% [11, 14]. In our study, all patients were treated as bridge to surgery with a significant improvement in symptoms and QoL before the surgical cancer resection.

SEMS placement has also been used since the 2000s to avoid emergency surgery for relief of obstructive symptoms [15] although a recent Cochrane review by Sagar [16] from five randomized trials showed that the use of colonic stents in malignant colorectal obstruction seems to carry no advantage over emergency surgery in terms of mortality and morbidity. However the author reported a significant benefit of SEMS placement in terms of hospital stay (11.53 versus 17.15 days), SEMS procedure/operating time (113.93 versus 143.85 minutes), and blood loss (50 mL versus 350 mL). A recent guideline on the management of obstructing cancer of the left colon showed that SEMS placement followed by elective surgery is more effective and cost efficient than emergency surgery [2].

Considering the adverse events, SEMS placement carries an overall complication rate of up to 25% [6]. Endoscopic technique and stent design, proximal colon stenting, obstruction from extracolonic malignancy, operator inexperience, and chemotherapy treatment can affect outcomes [17–22].

The new nitinol enteral uncovered stents by Taewoong (Seoul, Korea) are made of nitinol wire, which provides a flexible, fine mesh tubular conformable prosthesis which facilitates immediate, continuous wall apposition. Eight radiopaque markers allow an accurate release through the stricture. The final result is a uniform distension of the colonic wall, keeping the normal bowel anatomy and reducing the risk of SEMS decubitus and perforation. In the current study niti-S enteral uncovered stents (D-Type) placement has been effective in colonic decompression achievement in patients with stricturing CRC suitable for curative surgical resection. This bridge to surgery approach was effective and safe in all treated patients as an elective or emergency procedure. Some data are available on nitinol stents placement as palliative treatment [23], but, despite the small number of the enrolled patients, this is the only case series available in the literature on bridge to surgery treatment in operable patients.

In conclusion our prospective observational pilot study, though with the known limitations of this kind of study, showed that conformable nitinol SEMS can play an important role in the treatment of symptomatic CRC obstruction before curative surgery. This can be an effective and safe nonsurgical option also as rescue therapy. Further larger studies are needed to confirm the role of this kind of SEMS as bridge to surgery or palliative treatment evaluating also the clinical efficacy and/or adverse events associating chemotherapy treatment.

## Figures and Tables

**Table 1:** Patient demographic and clinical characteristics, and SEMS characteristics.

Characteristic	
Mean age, years (±SD)	69.2 ± 10.1
Male/female	7 (70%)/3 (30%)
Stenosis site:	
(i) Rectosigmoid colon	5 (50%)
(ii) Descending colon	2 (20%)
(iii) Splenic flexure colon	1 (10%)
(iv) Transverse colon	1 (10%)
(v) Ascending colon	1 (10%)
Mean stenosis length, cm (±SD)	3.6 ± 0.6 cm
Elective/emergency procedure	5 (50%)/5 (50%)
Patients who developed complications during followup	0 (0%)
Median time from stent placement to surgery, days (interquartile range)	16 (7–21)
